# The Ability to Acquire Iron Is Inversely Related to Virulence and the Protective Efficacy of *Francisella tularensis* Live Vaccine Strain

**DOI:** 10.3389/fmicb.2018.00607

**Published:** 2018-04-04

**Authors:** Joshua R. Fletcher, Deborah D. Crane, Tara D. Wehrly, Craig A. Martens, Catharine M. Bosio, Bradley D. Jones

**Affiliations:** ^1^Graduate Program in Genetics, University of Iowa, Iowa City, IA, United States; ^2^Immunity to Pulmonary Pathogens Section, Laboratory of Intracellular Parasites, Hamilton, MT, United States; ^3^Genomics Core, Research Technologies Branch, Rocky Mountain Laboratories, National Institute of Allergy and Infectious Diseases (NIAID), National Institutes of Health, Hamilton, MT, United States; ^4^Department of Microbiology, University of Iowa, Iowa City, IA, United States

**Keywords:** *Francisella tularensis*, iron acquisition mechanism, microbial pathogenesis, inflammatory mechanisms, virulence mechanisms, intracellular pathogen

## Abstract

*Francisella tularensis* is a highly infectious bacterial pathogen that causes the potentially fatal disease tularemia. The Live Vaccine Strain (LVS) of *F. tularensis* subsp. *holarctica*, while no longer licensed as a vaccine, is used as a model organism for identifying correlates of immunity and bacterial factors that mediate a productive immune response against *F. tularensis*. Recently, it was reported that two biovars of LVS differed in their virulence and vaccine efficacy. Genetic analysis showed that they differ in ferrous iron homeostasis; lower Fe^2+^ levels contributed to increased resistance to hydrogen peroxide in the vaccine efficacious LVS biovar. This also correlated with resistance to the bactericidal activity of interferon γ-stimulated murine bone marrow-derived macrophages. We have extended these findings further by showing that a mutant lacking bacterioferritin stimulates poor protection against Schu S4 challenge in a mouse model of tularemia. Together these results suggest that the efficacious biovar of LVS stimulates productive immunity by a mechanism that is dependent on its ability to limit the toxic effects of oxidative stress by maintaining optimally low levels of intracellular Fe^2+^.

## Introduction

*Francisella tularensis* is a highly infectious bacterial pathogen that parasitizes the cytosol of host cells and causes the lethal disease tularemia in humans. The low infectious dose and high morbidity and mortality associated with tularemia have led the United States Centers for Disease Control and Prevention to designate *F. tularensis* a Tier 1 Select Agent. Fear of the intentional misuse of *F. tularensis* has spurred research into understanding the pathogenesis of the organism and the development of a vaccine. No licensed vaccine for *F. tularensis* infection is currently available for widespread use, although, the *F. tularensis* Live Vaccine Strain (LVS) has, in recent years, been used to vaccinate laboratory staff in the US and in other countries and large numbers of individuals have been vaccinated in the past (Eigelsbach and Downs, 1961). LVS is an attenuated derivative of *F. tularensis* subsp. *holarctica* that was generated in the Soviet Union in the mid- twentieth century but an incomplete understanding of the genetic changes, and therefore the underlying mechanisms, leading to the attenuation of the strain have led to its discontinuation as a licensed vaccine. Although not available as a human vaccine, LVS is routinely used as a model to understand the genetic requirements of a *F. tularensis* strain to stimulate host immunity and as a tool to discover and characterize correlates of immunity in the host. Each of these areas of understanding are critical in the rational design of a future vaccine against virulent *F. tularensis* species.

Despite significant differences in virulence, members of the *Francisella* genus are very similar at the genetic level; many of the observed differences are genomic rearrangements and single nucleotide polymorphisms (SNPs) (Rohmer et al., 2006, 2007). The genome similarities of the *Francisella* strains have made identification of specific factors that mediate the high level of virulence displayed by Type A and Type B strains difficult, although genome comparisons between *F. tularensis* subsp. *tularensis* Schu S4 and less virulent LVS have identified some of the attenuating mutations. These mutations include fusion of the *fupA/B* genes and deletion of *pilA* (Lindgren et al., 2009). Another noted difference between the highly virulent Type A strains and the intermediately virulent type B strains (including LVS) is the levels of intracellular iron, with virulence being inversely correlated with iron levels (lower intracellular iron correlates with higher virulence and higher intracellular iron correlates with lower virulence) (Lindgren et al., 2011). Iron is an essential micronutrient for bacteria, and numerous studies have shown that it is highly sought after by bacterial pathogens, as host sequestration of iron (a part of nutritional immunity) can restrict the growth of numerous pathogens (Cassat and Skaar, 2013; Parrow et al., 2013). Apparently, the virulence strategy of *F. tularensis* represents an exception to this paradigm as Lindgren et al., found that higher virulence subspecies had lower levels of intracellular iron (Lindgren et al., 2011).

*Francisella* species import iron via siderophore-bound ferric iron via the Fsl and Fup systems, and ferrous iron via FeoB and FupA/B; the latter system is unique to LVS (Ramakrishnan et al., 2008; Thomas-Charles et al., 2013; Perez and Ramakrishnan, 2014; Ramakrishnan and Sen, 2014). The *feo* operon typically consists of *feoAB*, and less frequently *feoABC*; the *Francisella* chromosome encodes *feoA* and *feoB* separately, and lacks *feoC* (Cartron et al., 2006; Perez and Ramakrishnan, 2014). FeoB is a large transmembrane protein that has an N-terminal G-protein domain and a multi-pass transmembrane C-terminal domain (Marlovits et al., 2002; Andrews et al., 2003; Hantke, 2003). In other organisms, the Fe^2+^ import activity of FeoB requires interaction with the small protein FeoA, though the details of how FeoA stimulates FeoB activity are unknown (Su et al., 2010; Kim et al., 2012; Lau et al., 2013; Weaver et al., 2013). The general importance of iron in bacterial pathogenesis is reflected by the numerous ways that bacterial pathogens have evolved to obtain iron in a host, and several research groups have demonstrated that *feoB* contributes to or is required for full pathogenesis of *Salmonella, Campylobacter, Helicobacter, Legionella*, and others (Velayudhan et al., 2000; Robey and Cianciotto, 2002; Naikare et al., 2006; Aranda et al., 2009; Cassat and Skaar, 2013; Nagy et al., 2014). Multiple studies have characterized the Fe^2+^ uptake function of the *F. tularensis* FeoB and have linked this to its pathogenesis (Thomas-Charles et al., 2013; Perez and Ramakrishnan, 2014; Lindgren et al., 2015). However, it is not clear from these studies if the modulation of growth among *feoB* mutants is a direct result of differential ability to use and take up iron or if there are indirect effects of excess iron that impact bacterial replication. For example, while iron is necessary, excess Fe^2+^ can contribute to toxicity for an organism as ferrous iron can participate in the Fenton reaction with H_2_O_2_, poisoning iron-sulfur cluster enzymes in essential metabolic pathways (Imlay, 2013).

*Francisella tularensis* virulence is connected to the ability of the organism to avoid the detrimental effects of reactive oxygen species (ROS), such as H_2_O_2_ (Bou-Abdallah et al., 2002; Lindgren et al., 2007; McCaffrey et al., 2010; Lindemann et al., 2011; Crane et al., 2014; Ma et al., 2014). The bacterium couples glutamate metabolic pathways to H_2_O_2_ neutralization, and also maintains optimally low levels of intracellular Fe^2+^ such that Fenton reaction-mediated damage appears to be minimized (Lindgren et al., 2011; Ramond et al., 2014). When this optimal iron threshold is surpassed, numerous antioxidant enzymes are employed to protect the organism from toxic H_2_O_2_; mutants lacking enzymes like the Dyp peroxidase, superoxide dismutase and catalase are more sensitive to H_2_O_2_ damage, fail to inhibit host inflammatory signaling, and are often attenuated in murine infection models (Bakshi et al., 2006; Melillo et al., 2009, 2010; Llewellyn et al., 2011; Binesse et al., 2015; Rabadi et al., 2015; Shakerley et al., 2015). A growing body of literature has shown that *F. tularensis* utilizes numerous strategies to avoid activation of ROS-dependent host signaling pathways and killing by host-generated reactive oxygen and reactive nitrogen species (Buchan et al., 2009; Melillo et al., 2009, 2010; McKenna et al., 2010; Langmead and Salzberg, 2012; Binesse et al., 2015; Griffin et al., 2015; Rabadi et al., 2015; Shakerley et al., 2015). Furthermore, at least one phenotypic difference between the virulent *F. tularensis* Schu S4 strain and the non-pathogenic *F. novicida* strain is that the latter is considerably more sensitive to H_2_O_2_; this sensitivity is associated with activation of the AIM2 inflammasome (Zubay et al., 1972).

In this work we describe how regulation of iron uptake by *F. tularensis* LVS has a critical impact on the mouse virulence of the organism and on the resulting ability of the strain to induce effective protection against challenge with virulent *F. tularensis*. We first demonstrate how genetic variability in FeoB-mediated Fe^2+^ uptake observed in low vs. highly efficacious strains of LVS contributes to H_2_O_2_ sensitivity of *F. tularensis* LVS. We show that a LVS biovar that exhibits greater virulence in mice and engenders strong protective immunity possesses a single nucleotide polymorphism in the *feoB* gene that confers significantly higher resistance to H_2_O_2_ (due to lower iron acquisition) than other LVS biovars. In addition, we confirm that the modulation of iron uptake and the resulting sensitivity to H_2_O_2_ is not exclusive to *feoB*, but can be extended to other iron homeostasis systems in the bacterium. We previously identified a *F. tularensis* gene involved in iron homeostasis from a mutagenesis screen to identify genes important for growth in human monocyte derived macrophages (MDMs) (Salomonsson et al., 2009). This earlier work identified the bacterioferritin (*bfr*) gene as important for *F. tularensis* Schu S4 growth in MDMs infected with pools of transposon mutants (Salomonsson et al., 2009). Specifically, we demonstrate that deletion of bacterioferritin (*bfr*) increases sensitivity of the bacterium to H_2_O_2_, decreases virulence *in vivo*, and renders LVS poorly protective against challenge with virulent *F. tularensis*. Together, our findings connect the nutrient acquisition of LVS with its ability to provoke strong vaccine induced immunity.

## Materials and methods

### Mutant construction

Deletion of *feoB* (*FTL_0133*) and *bfr* (*FTL_0617*) genes was achieved by homologous recombination using derivatives of the non-replicating plasmid pJC84. Primer sequences are shown in Table 1. Briefly, upstream and downstream flanking DNA was amplified via PCR, and amplicons were cloned into the multiple cloning site of pCR2.1 (Life Technologies). A spectinomycin resistance cassette was cloned into the AvrII site in the 3′ end of each upstream PCR fragment. The upstream-spectinomycin resistance fragment was removed by digestion with AscI, and cloned into the AscI site in the 5′ region of the downstream PCR plasmid. The entire upstream-spectinomycin resistance-downstream fragment was cloned into the BamHI site of the suicide plasmid pJC84. Finally, the spectinomycin resistance cassette was removed by digestion with AvrII and the plasmid was re-ligated. Plasmids were electroporated into LVS (2.5 kV, 25 μF, and 600), and the bacteria were plated onto MMH agar with 50 μg/mL kanamycin after 2–3 h of outgrowth. Organisms were then grown overnight in broth lacking kanamycin, and were plated onto MMH agar with 8% sucrose for *sacB*-mediated counter-selection. Kanamycin sensitive colonies were screened by colony PCR to detect deletion of the *feoB* or *bfr* gene. Primers were designed so that they flanked the coding sequence of each gene such that an amplicon would be produced regardless of genotype.

### Bacterial strains and growth conditions

Plasmids and bacterial strains used in this work at listed in Tables 2, 3, respectively. Three different biovars of *F. tularensis* subsp. *holarctica* LVS were used in this work. LVS obtained from Karen Elkins (FDA, Bethesda, MD) and routinely utilized at the University of Iowa, here called Iowa LVS (University of Iowa) and the RML LVS and ATCC LVS, (Rocky Mountain Laboratories) (Su et al., 2007). Bacteria were routinely cultured on modified Mueller-Hinton (supplemented with 1% glucose, 0.025% ferric pyrophosphate, and 2% IsoVitaleX) agar or in broth, with 50 μg/mL of kanamycin or spectinomycin, as needed. Mueller-Hinton agar was also prepared without ferric pyrophosphate supplementation to assay for growth in lower iron conditions. For some experiments, bacteria were cultured in Chamberlain's defined medium with either 35 μM FeSO_4_ or 350 nM FeSO_4_, supplemented with antibiotics as needed. Agar plates were incubated at 37°C with humidity and 5% CO_2_ while broth cultures were grown at 37°C, shaken at 200 rpm.

**Table 1:** Primers used in this study.

5′feoB.up.asc1.bamh1–ggcgcgccggatccAGCATATCAAACACAAAGAAGAAGTATTG
3′feoB.up.avr2–cctaggAATCAGTTTTCAGGAGTTTATAGTATAG
5′feoB.down.asc1.avr2–ggcgcgcccctaggAATTCTAATTTGAATATACAGCTTTA 3′feoB.down.bgl2–agatctTTAGCATTTCACTAAGATTTGCACC
5′feoB.mut.check–AGATGATTGCTCAGTAATAACAGCAACTTTGC
3′feoB.mut.check–CATTAATAAGAATAGTATTCTCATTTTAAAATACCTC
5′feoB.SNP–AGACTTGCGATATTTTCAGTATTTGC
3′feoB.SNP–TTTACCGGCATATTCAAGTGCTGTGG
5′feoBc.kpn1–ggtaccATGAAATATGCTCTAGTTGGCAATCC
3′feoBc.sal1–gtcgacATATTTAAAGCTGTATATTCAAATTAG
5′feoAc.kpn1–ggtaccATGACTTATAACAAAAATGATAAATTTATTG
3′feoAc.sal1–gtcgacATATTTTTCGTAAACGCATCTTATAGCCTC
5′bferr.up.asc1.bamh1–ggcgcgccggatccTTTTAGTGATACTTTTTGAGACAATTGTCCC
3′bferr.up.asc1.avr2 – ggcgcgcccctaggCATATTGTTACCTCCATTATTTAAAACTCTAATC
5′bferr.down.asc1–ggcgcgccTAAAGGCTATTATCCTCGATGAGTTTTTCTTC
3′bferr.down.bamh1–ggatccAAGTATTTATCTGTAGTTACAATGGTGG
5′bfr.mut.check–ATATCATTTTTATTAAAATATCTAGGTTG
3′bfr.mut.check–ATAAATACTTTAAGTCACTAAATATCTCG

**Table 2:** Plasmids.

**Plasmids**	**Description**	**Source**
pCR2.1TOPO		Invitrogen
pGEMT Easy		Promega
pBB103	*Francisella-E. coli* shuttle vector, spect^R^	
pBB133	*Francisella-E. coli* shuttle vector containing the P*_*fslA*_-lacZ* reporter	Buchan et al., 2009
pJC84	*Francisella* suicide vector	Wehrly et al., 2009
pJF103	P_gro_-Iowa LVS *feoB* in pBB103, spect^R^	This study
pJF104	P_gro_-*feoB* D471Y in pBB103, spect^R^	This study
pJF109	Upstream and downstream *feoB* homology arms in pJC84	This study
pJF114	Upstream and downstream *bfr* homology arms in pJC84	This study
pJF118	P*_*gro*_-feoA* in pBB103	This study
pJF119	Iowa LVS *feoB* in pWKS30	This study
pJF120	*feoB* D471Y in pWKS30	This study

**Table 3:** Strains.

**Strains**	**Description or genotype**	**Source**
*F. tularensis* subsp. *holarctica* RML LVS		RockyMountain Laboratories
*F. tularensis* subsp. *holarctica* Iowa LVS		Karen Elkins, FDA
*F. tularensis* subsp. *holarctica* ATCC LVS		RockyMountain Laboratories
JF200	RML LVS carrying pJF103	This study
JF201	RML LVS carrying pJF104	This study
JF209	Iowa LVS Δ*feoB*	This study
JF212	JF209 carrying pJF103	This study
JF213	JF209 carrying pJF104	This study
JF297	RML LVS Δ*bfr*	This study
JF305	JF297 carrying pBB133	This study
*E. coli* Top 10	F- *mcrA* Δ(*mrr-hsd*RMS-*mcr*BC) Φ80*lac*ZΔM15 Δ *lac*X74 *rec*A1 *ara*D139 Δ(*araleu*)7697 *gal*U *gal*K *rps*L (StrR) *end*A1 *nup*G	Invitrogen
*E. coli* H1771	MC4100 *aroB feoB7 fhuF*::*plac* Mu	Kammler et al., 1993
JF280	pJF118 and pJF119 in *E. coli* H771	This study
JF281	pJF118 and pJF120 in *E. coli* H771	This study

### Murine infection

Eight to Ten week old female C57BL/6 mice were maintained on corncob bedding for 1 week prior to infection. Mice were intranasally infected with 25 μL of various doses of *F. tularensis* LVS strains and mutant derivatives that had been resuspended in PBS. Inocula were calculated by measuring the OD_600_ value of mid- to late-log phase grown organisms, and were confirmed by serial dilution onto modified Mueller-Hinton agar and enumeration after 2–3 days growth at 37°C with humidity, 5% CO_2_. Moribund animals (defined as having lost 25% of the initial body weight) were sacrificed in accordance with the protocol approved by the University of Iowa Institutional Animal Care and Use Committee.

### Genome sequencing

Genomic DNA from RML LVS and ATCC LVS was prepared from overnight cultures using DNeasy tissue kit (Qiagen) according to the instructions of the manufacturer. DNA was sequenced using an Illumina HiSeq2500 platform by the Institute for Genomic Medicine Genomic Center (University of California, San Diego, San Diego, CA), resulting in ~7 million reads per sample. Reads were trimmed for adapter sequence and trimmed and filtered for low quality bases using the FASTX-Toolkit (Hannon Lab, CSHL). Resulting reads were mapped to the LVS genome (NC_007880) using Bowtie2 (Ashkenazy et al., 2010). Analysis of genetic modifications, e.g., SNPs and gene deletions was performed using GATK comparing both RML LVS and ATCC LVS genomes to each other and the annotated LVS genome (Landau et al., 2005).

### Iron-regulated β-galactosidase reporter activity

To assess iron uptake-regulated gene expression, Miller assays were performed with Iowa LVS, RML LVS, and ATCC LVS carrying the *fslA* promoter-*lacZ* fusion on the pBB103 plasmid (Troxell and Hassan, 2013). Bacteria were grown in Chamberlain's defined medium (supplemented with 50 μg/mL spectinomycin) overnight, and β-galactosidase activity was assayed using the standard Miller assay (Sullivan et al., 2006).

### FeoB function in a heterologous reporter system

The coding sequence of *feoB* was PCR-amplified from both Iowa and RML LVS using the high fidelity Phusion polymerase (New England Biolabs). Sanger sequencing was performed to confirm that the SNP observed in the RML LVS background was present. The *feoA* coding sequence was cloned into the KpnI/SalI sites of a derivative of pTrc99a, immediately downstream of the *groE* promoter. The entire P_*groE*_-*feoA* fragment was removed by digestion with BamHI and SalI, and ligated into the same sites in pBB103. Both this plasmid and the pWKS30 containing *feoB* were transformed into the *E. coli* H1771 strain (MC4100 *aroB feoB7 fhuF*::*plac* Mu). Control strains were also generated that carried only one of the plasmids. Miller assays were performed to measure FeoB Fe^2+^ import activity via the readout of Fur repression of *fhuF::placZ*. All strains were grown in LB supplemented with 50 μg/mL of spectinomycin and 100 μg/mL of ampicillin, as necessary.

### Hydrogen peroxide sensitivity

To measure resistance to H_2_O_2_-mediated killing, bacteria were grown in modified Mueller-Hinton broth and mid- to late-log phase organisms were pelleted at 13,200 RPM for 5 min, washed in PBS, and ~10^6^ CFU were resuspended in 200 μL PBS with or without 100 μM hydrogen peroxide (H_2_O_2_) in a 96-well dish. The samples were incubated at 37°C with humidity and 5% CO_2_ for 1 h. The culture from each well was then serially diluted in sterile PBS, plated onto modified Mueller-Hinton agar (with antibiotic when necessary) and the number of surviving organisms for each strain was enumerated after 2–3 days.

### *In vitro* infections

LVS infections of bone marrow derived macrophages (BMM) were performed as previously described (Su et al., 2007). Briefly, BMM were differentiated from femurs of C57Bl/6 mice over the course of 7 days in complete DMEM (cDMEM, DMEM supplemented with 10% heat-inactivated fetal calf serum [FCS], 0.2 mM l-glutamine, 1 mM HEPES, and 0.1 mM non-essential amino acids [NEAA], all from Life Technologies) supplemented with M-CSF (Peprotech). Immediately prior to infection medium was removed and reserved for addition after infection. The indicated bacterial strains were added at a MOI of 50 and co-incubated with BMM for 90 min. Bacteria were then removed and fresh medium containing 50 μg/ml of gentamicin was added to kill extracellular organisms. After 45 min, BMM were washed extensively with D-PBS (Life Technologies) and the reserved medium was returned to the wells. IFN-γ (Peprotech) was then added to the indicated groups at a final concentration of 10 U/ml. At the indicated time points, medium was collected and assessed for cytokines as described below. BMM were washed 3 times with D-PBS followed by lysis with water. Lysates were serially diluted and plated on to MMH agar for enumeration of viable organisms.

### Statistics

All statistics were calculated with Graphpad Prism software (San Diego, CA). To compare ß-galactosidase activity across multiple strains we used one-way ANOVA with Dunnett's multiple comparisons test. For comparisons of multiple strains across multiple conditions, we utilized two-way ANOVA with either Sidak's or Tukey's multiple comparisons tests. When applicable, an unpaired two-tailed *t*-test was used. The LD_50_ values were calculated using the method of Reed and Muench. To compare survival after *F. tularensis* SchuS4 challenge, a log-rank sum test was utilized. The error bars in the presented data represent the standard error of the mean. A *p* < 0.0001 is represented by ^****^p < 0.01 is represented by ^**^p < 0.05 is represented by ^*^, and “ns” indicates “not significant.”

## Results

### Genome sequencing of *F. tularensis* LVS strain

It was recently reported that there are differences in virulence and vaccine efficacy between LVS isolates (Deng et al., 2006; Su et al., 2007). Specifically, a low passage strain, termed RML LVS, had increased virulence in C57Bl/6 mice but also engendered complete protection against challenge with virulent *F. tularensis* subsp *tularensis*. In contrast, LVS strain ATCC29684 (ATCC LVS) had lower virulence in C57Bl/6 mice and failed to protect animals from lethality following *F. tularensis* challenge. The specific genetic changes that could account for the dramatic *in vivo* differences between these two strains were not identified. Therefore, in this work we have sequenced the genomes of these two LVS biovars to identify and characterize these genetic differences. Surprisingly, we detected very few differences in the sequences between RML LVS and ATCC LVS. We detected a 93 base pair deletion in the gene encoding a Dyp-type peroxidase that has been previously characterized (Binesse et al., 2015). Additionally, we detected a single nucleotide polymorphism in the *feoB* gene encoded by RML LVS. The C to A substitution leads to an aspartate to tyrosine mutation in the coding sequence of *feoB*. The D471Y mutation maps to a cytoplasmic loop of the FeoB protein, adjacent to a highly conserved glycine residue that is predicted to be functionally important by the ConSurf program (Kammler et al., 1993; Buchan et al., 2008). Although the genome of the lab stock of Iowa LVS from the Jones lab has not been completely sequenced, the strain was also included in the experiments described here. Sanger sequencing confirmed that the Iowa LVS *feoB* encodes an aspartate at residue 471, similar to the published LVS genome. FeoB is an inner membrane protein that imports ferrous iron into the bacterial cytoplasm and has previously been linked to virulence as one of two major iron uptake pathways in LVS (Schulert et al., 2009; Thomas-Charles et al., 2013; Perez and Ramakrishnan, 2014). The substitution of a large, hydrophobic tyrosine for an aspartate at residue 471 led us to hypothesize that the FeoB protein encoded by the RML LVS allele imports Fe^2+^ poorly, or not at all. We determined if differences in iron acquisition could account for the varied virulence and vaccine efficacy of RML LVS vs. ATCC LVS. Since iron plays an integral role in host-induced oxidative stress, it is likely that controlling intracellular iron levels is a significant component of the virulence strategy of *F. tularensis*. Furthermore, sensitivity to oxidative stress may contribute to the vaccine efficacy of LVS given that the ATCC biovar of LVS has an aspartate at residue 471 in FeoB and the 93 base pair *dyp* deletion, and is both relatively attenuated in murine infections and stimulates a weak immune response against a challenge with virulent *F. tularensis* Schu S4 (Griffin et al., 2015).

### RML LVS displays phenotype consistent with lower intracellular Fe^2+^ than the ATCC LVS or Iowa LVS

**Figure 1:**
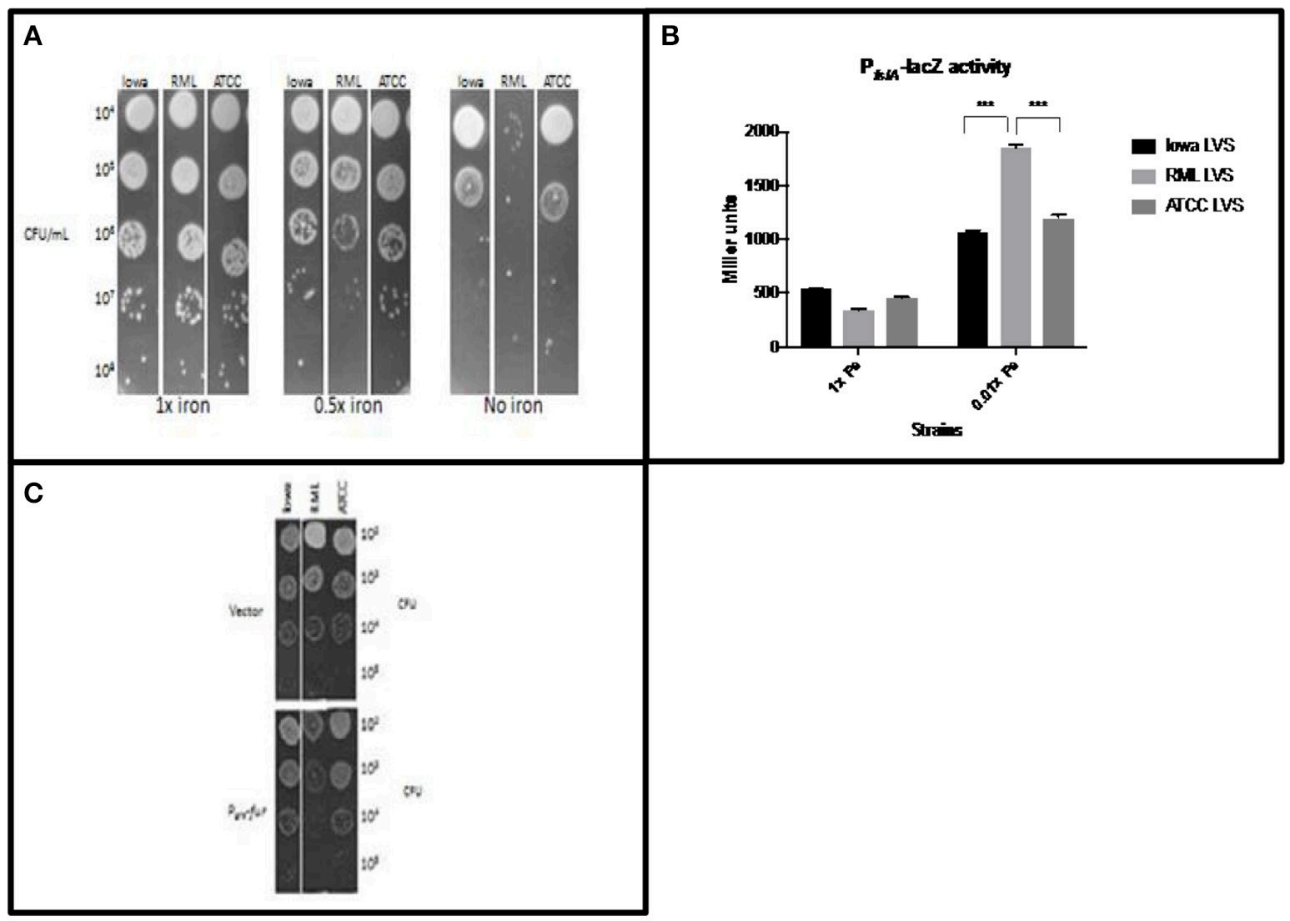
RML LVS has relatively less intracellular iron than other LVS biovars. **(A)** Bacteria were grown overnight in standard MMH broth, serially diluted in sterile PBS and plated for enumeration on MMH agar with 0.025%, 0.0125%, or no added Fe^3+^PP_i_. Data from a representative experiment is shown. **(B)** Miller assay for each LVS carrying a plasmid with the Fur regulated P_*fslA*_*-lacZ* reporter. Bacteria were grown overnight in Chamberlain's defined medium with 35 μM (high iron) or 350 nM (low iron) FeSO_4_. Two-way ANOVA with Sidak's multiple comparisons was used to test for significance. Shown is a representative Miller assay from replicate experiments. **(C)** Strains overexpressing *fur* were grown in MMH broth overnight, serially diluted into sterile PBS and plated on standard MMH agar. Shown are representative data from two similarly designed experiments.

Our first set of experiments was designed to test the hypothesis that RML LVS has less intracellular iron than either the Iowa LVS or the ATCC LVS. Strains were grown overnight in standard modified Mueller-Hinton (MMH) broth, serially diluted in PBS, and ten-fold dilutions of liquid bacterial cultures were spotted onto MMH agar with varying concentrations of iron (Figure 1A). Growth was identical among each strain when spotted onto the control agar containing the typical concentration of iron (0.0025% ferric pyrophosphate) routinely used for propagation of *F. tularensis*. When the iron concentration was reduced by 50%, both the Iowa LVS and ATCC LVS had growth patterns similar to that observed on the control plate, however, RML LVS exhibited growth restriction at this concentration of iron. Fewer isolated colonies were observed and lawn growth was less luxurious when compared to the Iowa and ATCC LVS. On MMH agar lacking added iron, all three strains exhibited growth restriction, however the reduced growth phenotype of RML LVS was exacerbated, with extremely poor growth even at the lowest dilution plated. Iowa and ATCC LVS both grew as lawns at these dilutions, indicating that they were able to scavenge sufficient iron from the agar plate environment, while RML LVS could not. The decrease in RML LVS growth was approximately two orders of magnitude greater than that observed for either the Iowa or ATCC LVS, consistent with the hypothesis that RML LVS has less intracellular iron under conditions of iron limitation. We interpreted these results to mean that RML LVS had inherently lower levels of intracellular iron and that when growing the strain on agar with less iron, the amount that RML LVS could acquire for growth was limiting, thus the strain grew poorly relative to the Iowa LVS or ATCC LVS.

To confirm that the mutation carried in *feoB* by RML LVS had a direct consequence on iron levels, we performed experiments designed to give a semi-quantitative measure of intracellular iron levels. To avoid toxicity associated with the Fenton reaction that include lipid peroxidation, DNA damage, and poisoning of the iron-sulfur cluster enzymes of essential metabolic pathways, bacteria have evolved regulatory mechanisms to maintain iron homeostasis. One such example is the Fur system (Troxell and Hassan, 2013). The Fur transcriptional repressor is an allosteric regulator that uses Fe^2+^ as a co-repressor; when iron levels are sufficient, the Fur-Fe^2+^ complex binds to Fur Box sequence motifs in the promoters of iron uptake genes to mediate their repression. When iron is limiting, less Fe^2+^ is bound to Fur, decreasing its ability to bind DNA. This leads to de-repression of iron uptake genes. Since several studies have demonstrated that *Francisella* has a Fur regulatory system that functions similarly to other organisms (Deng et al., 2006; Sullivan et al., 2006; Buchan et al., 2008; Ramakrishnan et al., 2008), we were able to design experiments to indirectly measure *F. tularensis* LVS intracellular iron levels. A plasmid carrying a Fur-regulated P_*fslA*_*-lacZ* construct was introduced into the RML LVS, Iowa LVS, and ATCC LVS. Iron limitation is known to relieve Fur repression at the *fslA* promoter, so increased P_*fslA*_*-lacZ* reporter activity correlates with decreasing concentrations of intracellular Fe^2+^ (Buchan et al., 2009). Each LVS strain was grown in Chamberlain's defined medium (CDM) with 35 μM (high iron) or 350 nM (low iron) FeSO_4_, and β-galactosidase activity was measured after overnight growth (Figure 1B). As predicted, iron starvation induced high expression of P_*fslA*_*-lacZ* in all three strains; however, the *lacZ* reporter expression was approximately 2-fold higher in RML LVS than that observed for Iowa LVS or ATCC LVS. The higher activity of the P_*fslA*_*-lacZ* construct in the RML LVS is consistent with the hypothesis that this strain has lower levels of intracellular Fe^2+^ when iron is limiting in the growth medium.

To assess intracellular iron levels in RML LVS by another method, we reduced the ability of each strain to import iron by overexpression of Fur (which downregulates genes encoding iron uptake systems). The *F. tularensis fur* gene was placed under the control of the *groE* promoter, and the construct was introduced into each strain. Bacteria were grown in MMH broth with standard iron concentrations overnight (no differences in growth rate were observed between the two strains in iron replete conditions), serially diluted in PBS and plated onto standard MMH agar (Figure 1C). Iowa LVS and ATCC LVS, when overexpressing Fur, exhibited no significant difference in colony growth compared to vector controls, indicating that intracellular pools of iron were still sufficiently high to support normal growth, even with Fur overexpression. In contrast, RML LVS had much smaller colony size and poor lawn formation. We interpret these results to indicate that Fur overexpression in RML LVS had a greater impact on growth because Fur repression of iron uptake genes decreased the intracellular Fe^2+^ concentration to levels such that the strain could not grow normally, even though sufficient iron was present in the agar media. These results are consistent with the hypothesis that intracellular Fe^2+^ pools of RML LVS are lower than in Iowa LVS or ATCC LVS and can be manipulated to become limiting for growth in conditions that are not limiting for the other two strains.

### FeoB D471Y does not complement *E. coli ΔfeoB fhuf*::λ*plac*

**Figure 2:**
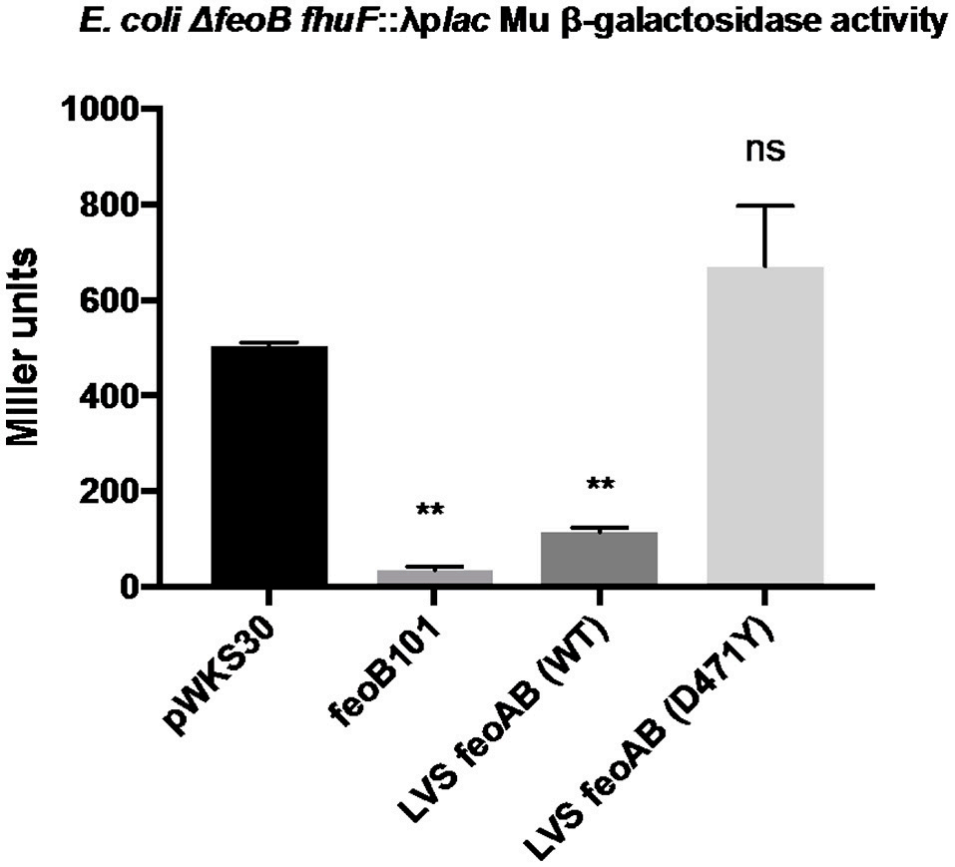
The RML LVS *feoB* allele is non-functional in a heterologous iron reporter system. The coding sequence of each *feoB* allele was cloned into the low copy plasmid pWKS30 and introduced to *E. coli* H1771. The coding sequence of *feoA* was fused to the *groE* promoter in a derivative of plasmid pBB103 and supplied to *E. coli* H1771 in tandem with the *feoB* expression plasmids, and Miller assays were performed as described in the Materials and Methods. A one-way ANOVA with Dunnett's multiple comparisons test was used to test for significance against the control pWKS30 plasmid. Significance was set at *p* < 0.05. ^**^Indicates a significant difference between the labeled samples and pWKS30. Shown is a representative experiment from three replicates.

The data demonstrating that RML LVS has lower intracellular pools of iron than either Iowa LVS or ATCC LVS suggest that the single nucleotide change in the *feoB* gene of the RML strain encodes a protein with significantly reduced Fe^2+^ import activity. To assess the ability of the FeoB D471Y protein to transport iron, we made use of a well-characterized *E. coli* iron reporter strain that lacks *feoB* (Kammler et al., 1993; Weaver et al., 2013). This strain, *E. coli* H1771, has a chromosomal transcriptional *lacZ* reporter in the Fur-regulated gene *fhuF* which can be used to report the relative intracellular iron concentrations within the strain. When Fe^2+^ levels are high, expression of the *fhuF*::*lacZ* reporter is low and when Fe^2+^ levels are low, high β-galactosidase activity is observed. The *feoB* alleles from Iowa LVS and RML LVS (D471Y) were introduced into *E. coli* H1771 on the low copy number plasmid pWKS30, and Miller assays were performed (Figure 2). Initial experiments supplying only *feoB* (either allele) showed no repression of *fhuF::lacZ*, indicating that FeoB alone is not sufficient for Fe^2+^ import in this reporter system (data not shown). In most bacterial species encoding a *feo* system, the small *feoA* gene is encoded either in an operon with *feoB* or elsewhere on the chromosome and the encoded protein is required to stimulate FeoB Fe^2+^ import activity (Cartron et al., 2006; Lau et al., 2013; Weaver et al., 2013). When the *Francisella feoA* was supplied on pBB103 in concert with *feoB* (Iowa/ATCC allele) on pWKS30, significant repression of the *fhuF*-*lacZ* reporter was observed (~ 80% reduction in expression relative to the empty vector control), indicating that Fe^2+^ import was significantly increased. In contrast, complementation of H1771 with *feoA* and *feoB* D471Y (RML allele) failed to repress *fhuF::lacZ*. This result is consistent with the hypothesis that the FeoB D471Y protein from the RML LVS is significantly impaired in its ability to import Fe^2+^.

### The *feoB* d471Y allele is associated with resistance to H_2_O_2_

**Figure 3:**
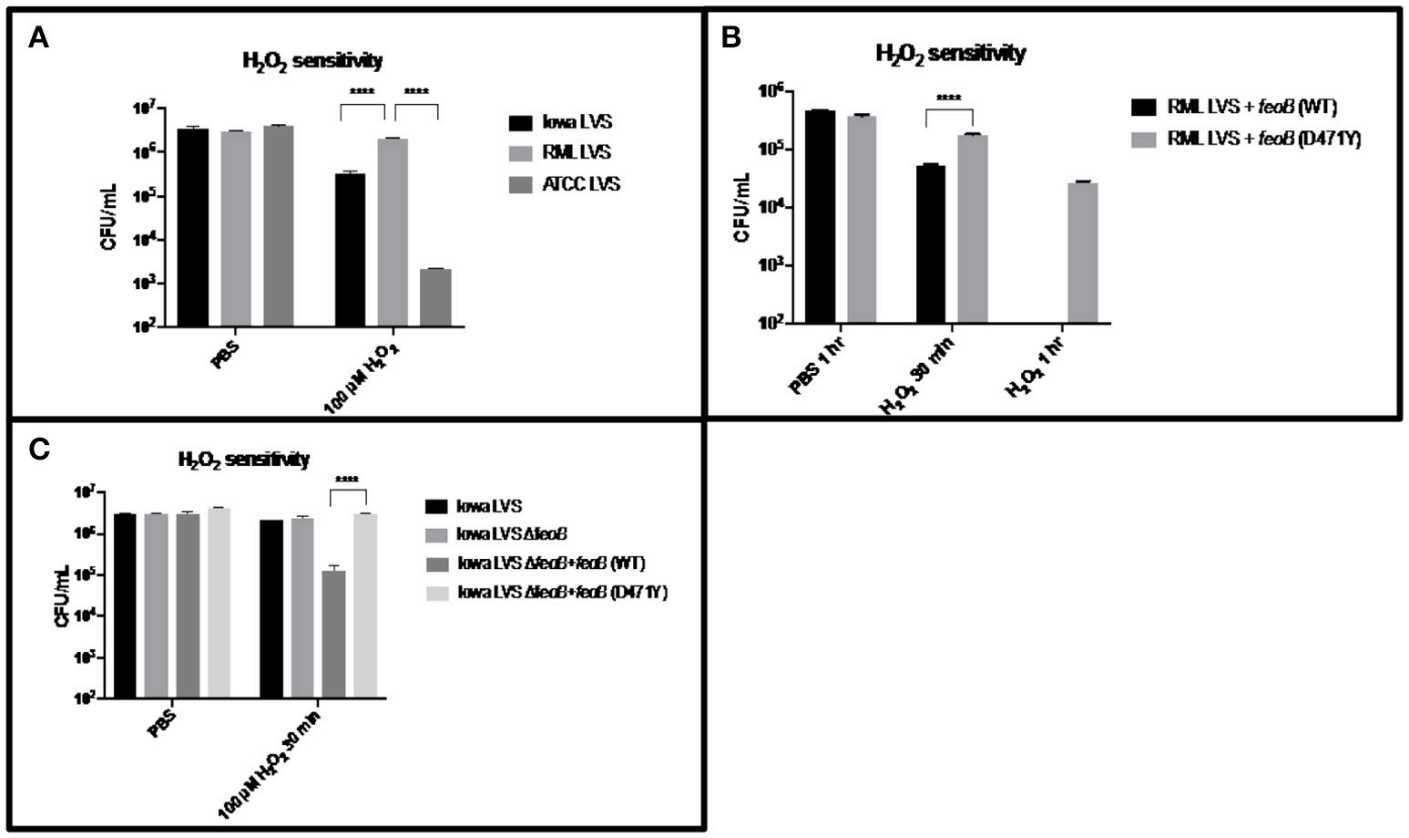
RML LVS is more resistant to H_2_O_2_ as a result of the point mutation in its *feoB*. **(A)** Strains were grown overnight in MMH broth, resuspended to ~10^6^ CFU in PBS and exposed to 100 μM H_2_O_2_ for 1 h at 37°C. Samples were serially diluted in sterile PBS and plated in triplicate. Shown is the CFU/ml in PBS alone and PBS + H_2_O_2_ from a representative experiment. A two-way ANOVA with Tukey's multiple comparisons test was used to test for significance. Shown is a representative of 3 replicates. **(B)** Each *feoB* allele was constitutively expressed from the *groE* promoter in a derivative of the multi-copy plasmid pBB103. These plasmids were introduced into the RML LVS and H_2_O_2_ sensitivity assays were performed as before, with the addition of a 30-min time point. Shown is the CFU/ml in PBS alone and PBS + H_2_O_2_ from a representative experiment. A two-way ANOVA with Sidak's multiple comparison test was used to test for significance. Shown is a representative of two replicates. **(C)** An Iowa LVS Δ*feoB* mutant was complemented with each *feoB* allele *in trans*, and H_2_O_2_ sensitivity assays were performed as before. A two-way ANOVA with Tukey's multiple comparisons test was used to test for significance. Significance was set at *p* < 0.05. ^****^Indicates a significant difference between the two samples bracketed. Shown is a representative of two replicates.

Since we have provided several lines of evidence consistent with the hypothesis that RML LVS has less intracellular Fe^2+^ than either the Iowa or ATCC LVS strains and Lindgren *et al*. demonstrated that strains with lower intracellular iron concentrations are more resistant to H_2_O_2_, we hypothesized that RML LVS would be more resistant to killing by H_2_O_2_ than Iowa LVS or ATCC LVS. Bacteria were exposed to PBS alone or PBS with 100 μM H_2_O_2_ in a 96-well dish for 1 h at 37°C with 5% CO_2_ and humidity. Following incubation, the bacteria were serially diluted in PBS and plated onto MMH agar (Figure 3A). The RML LVS was typically ten-fold more resistant to H_2_O_2_ than the Iowa strain, and approximately 100-fold to 1,000-fold more resistant than ATCC strain. The genome of ATCC encodes a Dyp peroxidase with a 93-base pair deletion, and it has been reported that this deletion can contribute to increased sensitivity to H_2_O_2_; an Iowa LVS Δ*dyp* mutant matches the H_2_O_2_ sensitivity of ATCC LVS (data not shown) and is in agreement with the data from Binesse et al. (2015).

Given the increased sensitivity of Iowa LVS to H_2_O_2_, and the functional complementation of an *E. coli feoB* mutant by the Iowa *feoB* allele, we next tested if the RML LVS could be sensitized to H_2_O_2_ by increasing the intracellular Fe^2+^ pool via overexpression of a functional *feoB*. This was achieved by transforming the strain with a plasmid carrying constructs P_*gro*_*-feoB* (Iowa/ATCC allele–high Fe^2+^ transport) and P_*gro*_*-feoB* (D471Y; RML allele–low Fe^2+^ transport) which both overexpress *feoB*. H_2_O_2_ sensitivity assays were performed as described previously, except that a 30 min H_2_O_2_ exposure time point was also included in case overexpression of the *feoB* genes led to substantially faster killing by hydrogen peroxide (Figure 3B). Nearly one log of killing was observed at 30 min for the RML + P_*gro*_*-feoB* (Iowa/ATCC), while RML + P_*gro*_*- feoB* (D471Y) only had a two-fold reduction in viability. The effect was significantly more pronounced at the 1 h time point, with no viable bacteria recovered at the level of detection (10^2^ CFU) from RML + P_*gro*_*-feoB* (Iowa/ATCC). In contrast, over 10^4^ CFU were recovered at 1 h from the RML + P_*gro*_*- feoB* (D471Y). These data demonstrate that the single nucleotide change in the *feoB* gene (D471Y0 encoded in the RML LVS genome results in significantly higher resistance to H_2_O_2_ compared to the *feoB* allele in either Iowa LVS or ATCC LVS.

To confirm that the H_2_O_2_ resistance phenotype conferred by *feoB* D471Y is not unique to the RML genetic background, we constructed a Δ*feoB* mutant in the Iowa LVS background and complemented it with the P_*gro*_*-feoB* constructs. The Iowa LVS strain background was chosen over the ATCC LVS background, as the latter strain was approximately 100-fold more sensitive to H_2_O_2_ than the RML LVS due to the 93 base pair deletion in the *dyp* peroxidase gene, while RML LVS and Iowa LVS both encode a full length *dyp*. H_2_O_2_ sensitivity assays were performed as described previously and, consistent with results in the RML strain background, overexpression of *feoB* D471Y mediated significant resistance to H_2_O_2_ in the Iowa LVS Δ*feoB* mutant, relative to that when the Iowa/ATCC *feoB* was overexpressed (Figure 3C).

### Bacterioferritin protects against hydrogen peroxide

**Figure 4:**
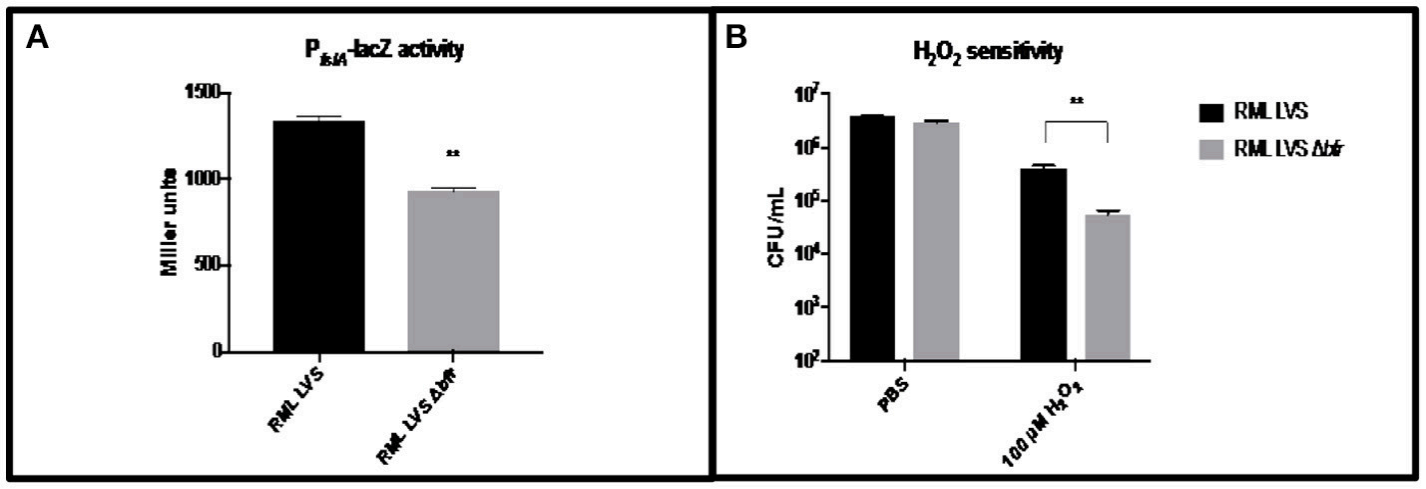
Iron related phenotypes of RML LVS Δ*bfr*. **(A)** P_*fslA*_*-lacZ* reporter activity is decreased in the RML LVS relative to wild type. Welch's *t*-test was used to test for significance. Shown is a representative of five replicates. **(B)** The RML LVS Δ*bfr* is more sensitive to H_2_O_2_ killing than the wild type. A two-way ANOVA with Sidak's multiple comparisons test was used to test for significance. Significance was set at *p* < 0.05. ^**^Indicates a significant difference between the samples indicated. Shown is a representative of four replicates.

To assess the role of the *bfr* gene in *F. tularensis* iron homeostasis and oxidative stress resistance, a Δ*bfr* strain was constructed in the RML LVS and P_*fslA*_*-lacZ* assays, as well as H_2_O_2_ sensitivity assays were performed as described above. No growth differences were observed in the Δ*bfr* mutant compared to the wild type strain. The RML LVS Δ*bfr* strain exhibited a modest decrease (~20%) in P_*fslA*_*-lacZ* reporter activity relative to the parent strain (Figure 4A), while in the H_2_O_2_ sensitivity assay, as before, the RML LVS exhibited relatively high level resistance to H_2_O_2_. The Δ*bfr* mutant had ~10-fold fewer viable organisms, similar to Iowa LVS (Figure 4B). A possible explanation for these results is that in the absence of the Bfr iron storage protein, intracellular ferrous iron levels are elevated relative to the parent RML LVS, which may lead to increased ferrous iron available for the Fenton reaction in the presence of H_2_O_2_ and the increased toxicity observed in the assay. Consistent with this, P_*fslA*_*-lacZ* activity was modestly reduced (~20%) in the RML LVS Δ*bfr* mutant (Figure 4B). As a result, we conclude that bacterioferritin contributes to protection against oxidative stress, consistent with bacterioferritin function in *Neisseria gonorrhea* and *Mycobacterium tuberculosis*.

### Expression of the Iowa/ATCC LVS *feoB* allele is deleterious in IFN-γ-stimulated macrophages

**Figure 5:**
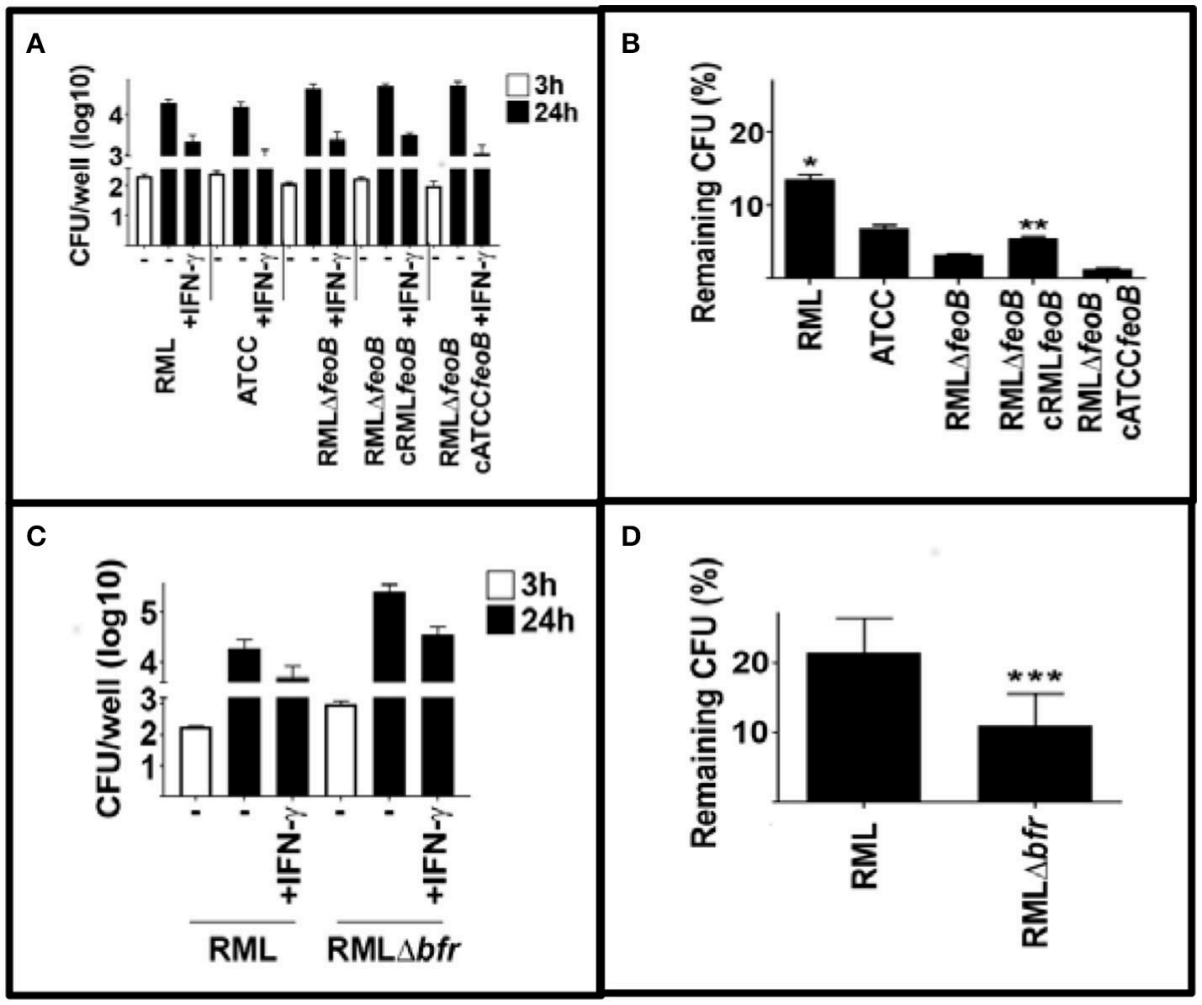
Mutations in iron acquisition enhance sensitivity of LVS to IFN-γ mediated killing. BMM were infected with the indicated strains of LVS and were treated with IFN-γ (10 U/ml) immediately after infection. Three and 24 h after infection intracellular bacteria were enumerated. **(A,B)** RML LVS is more resistant to IFN-γ mediated killing compared to all other strains tested. Deletion of the *feoB* gene rendered RML LVS more sensitive to IFN-γ mediated killing and complementation with homologous *feoB*, but not ATCC *feoB* partially restored resistance to IFN-γ. **(C,D)** Deletion of the bacterioferritin (*bfr*) also increased RML LVS sensitivity to IFN-γ mediated killing. Error bars represent SD. Significance was tested via two-way ANOVA followed by Tukey's multiple comparison of means. Significance was set at *p* < 0.05. ^*^, significantly greater than all other samples. ^**^, significantly greater than RMLΔ*feoB*. ^***^, significantly less than RML LVS. Data is representative of three experiments of similar design.

One mechanisms by which IFN-γ can control intracellular bacteria is to provoke production of reactive nitrogen and reactive oxygen species. It has been reported that IFN-γ mediated control of *Francisella* replication can be independent of RNS and ROS (Edwards et al., 2010). However, this may be due to its ability to limit toxicity of ROS by restricting iron. Therefore, we hypothesized that strains of LVS that were unable to control iron acquisition similarly to wild type RML LVS would be more sensitive to host cell IFN-γ mediated killing. We first compared RML LVS, ATCC LVS, RML LVS Δ*feoB* and RML LVS overexpressing RML *feoB* or Iowa/ATCC *feoB*. In agreement with our hypothesis RML LVS was the least sensitive to IFN-γ mediated killing among the strains tested (Figures 5A,B). Deletion of the *feoB* gene rendered the bacteria significantly more sensitive to IFN-γ mediated killing (Figures 5A,B). However, resistance to IFN-γ was partially restored in bacteria complemented with RML *feoB*. Complementation with ATCC *feoB* did not significantly change RML LVS sensitivity to IFN-γ mediated killing. We next tested the ability of RML LVSΔ*bfr* to resist IFN-γ mediated killing. Similar to RML LVS Δ*feoB*, RML LVS Δ*bfr* was significantly more sensitive to IFN-γ mediated killing compared to wild type controls (Figures 5C,D). Together these data suggest that alterations in ferrous iron homeostasis confer sensitivity to IFN-γ mediated control of intracellular replication of LVS.

### RML LVS Δ*bfr* is attenuated in murine infection and fails to protect against *F. tularensis* SchuS4

**Figure 6:**
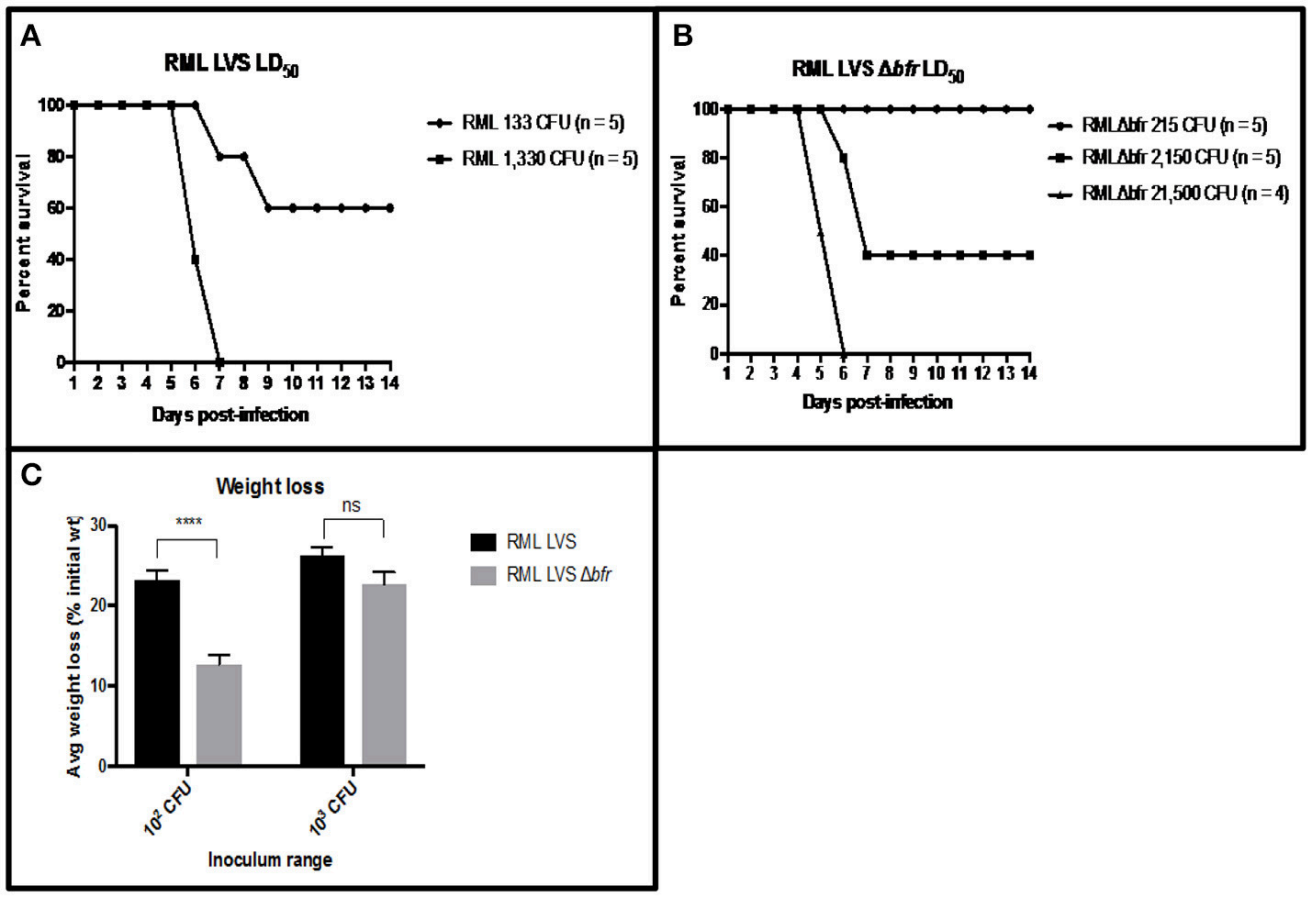
Bacterioferritin is required for optimal RML LVS virulence and. **(A)** Groups of mice (*n* = 5) were intranasally infected with 25 μl inocula containing 133 or 1,330 CFU of RML LVS in PBS. Mice were weighed daily and humanely sacrificed after weight loss ≥ 25% of their initial weight. LD_50_ values were calculated via the method of Reed and Muench (1938). **(B)** Groups of mice (*n* = 5) were intranasally infected with 25 ul inocular containing 215, 2,150 or 21,500 CFU of RML LVS Δ*brf* in PBS. Mice were weighed daily and humanely sacrificed after weight loss ≥ 25% of their initial weight. LD_50_ values were calculated via the method of Reed and Muench (1938). **(C)** The average weight loss of mice infected with 10^2^ or 10^3^ of the RML LVS strain or 10^2^ or 10^3^ of the RML LVS Δ*bfr* is represented by the bars in the graph. The significance of the weight loss of the mice infected with the RML LVS strain was compared to the weight loss of the mice infected with the RML LVS Δ*bfr* strain Mice infected with RML LVS were closer to or at clinical endpoint at all doses, while mice infected with RML LVS Δ*bfr* lost significantly less weight at the lowest infectious dose. Significance was set at *p* < 0.05. ^****^Indicates a significant difference between the two samples bracketed. Significance was tested via log-rank sum test.

As strains with increased sensitivity to H_2_O_2_ are associated with IFNγ-mediated killing in BMMs, we sought to determine if strains with increased sensitivity to H_2_O_2_ *in vitro* might also be attenuated in a mouse model of respiratory tularemia. Disappointingly, several efforts to assess virulence of Iowa LVS, its isogenic Δ*feoB* mutant and complemented derivatives gave inconclusive results, likely due to plasmid instability in the LVS strains in the absence of antibiotic selection *in vivo*. Thus, we pursued an alternative approach to assay for the role of altered ferrous iron homeostasis *in vivo* and the ability of RML LVS to provide protection against a challenge with *F. tularensis* SchuS4. To this end, we used the H_2_O_2_ sensitive RML LVS Δ*bfr* mutant in murine intranasal infections. RML LVS was previously reported to have a LD_50_ of 174 CFU in C57BL6 mice (Griffin et al., 2015). We confirmed this value by performing a similar infection experiment and calculated a LD_50_ value for RML LVS in C57BL/6 mice of 195 CFU (Figure 6A). To test if bacterioferritin contributed to the virulence of the *F. tularensis* RML LVS, three groups of mice (*n* = 5 per group) were intranasally infected with 215 CFU, 2.15 × 10^3^, or 2.15 × 10^4^ CFU of the RML LVS Δ*bfr* mutant, and their weight and health status was followed daily over the course of the experiment. All mice that received 215 CFU survived (average weight loss of ~12 percent) while forty percent of the mice survived infection with 2 × 10^3^ CFU (average weight loss of ~22%); none survived 2 × 10^4^ CFU. Importantly, it took >2,000 CFU of the RML LVS Δ*bfr* strain to yield survival curves and weight losses in mice that approximated that observed for mice infected with only 133 CFU of RML LVS, a >15-fold difference in inoculum (Figures 6B,C). The estimated LD_50_ of the RML LVS Δ*bfr* mutant was 1,464 CFU. These results demonstrate that the bacterioferritin protein contributes to the virulence of RML LVS in mice; however, future work is needed to determine if this is a consequence of the H_2_O_2_ sensitivity of this mutant.

**Figure 7:**
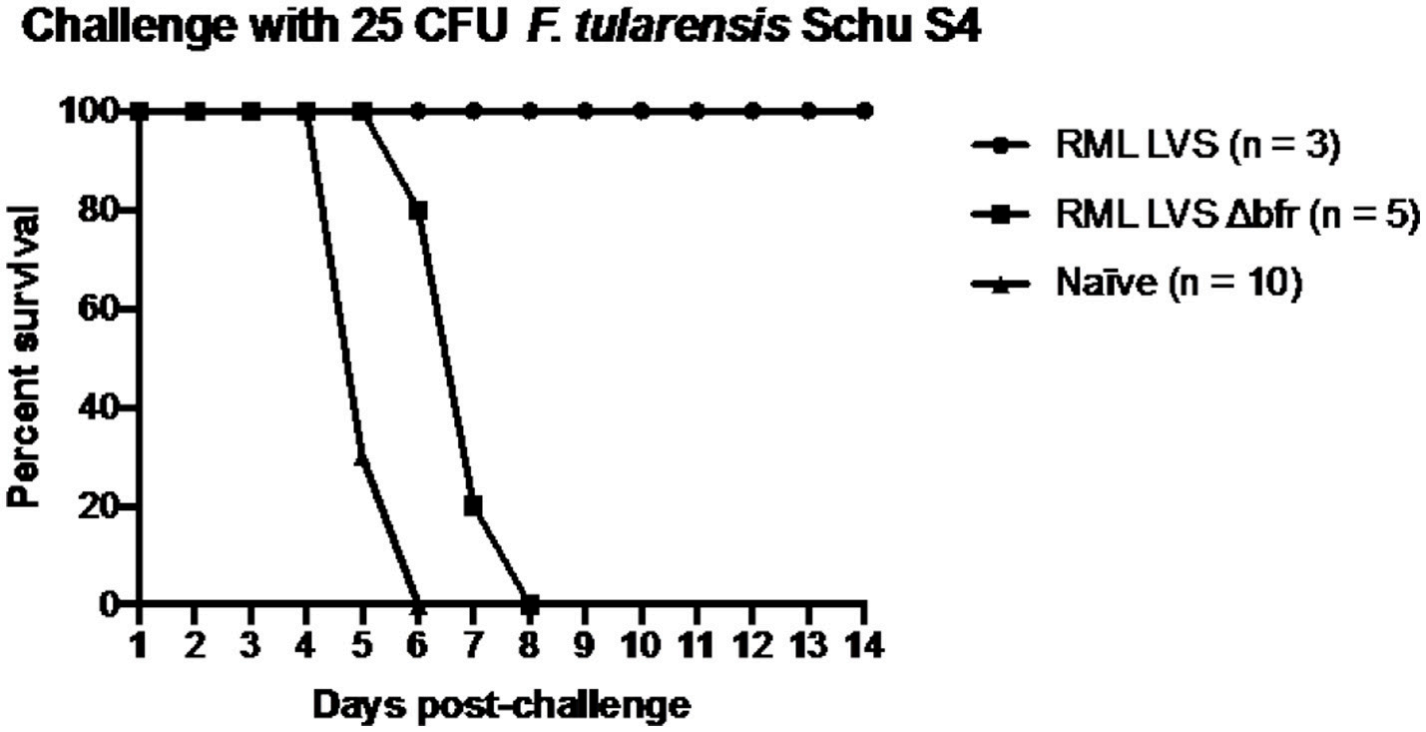
RML requires bacterioferritin to elicit protection against an intranasal challenge of 25 CFU *F. tularensis* Schu S4. Mice were infected with either 121 CFU of RML LVS or 215 CFU of the bacterioferritin mutant and allowed to recover for fix weeks before challenge. The challenge inoculum was suspended in 25 μl sterile PBS and administered intranasally. Health was monitored for the duration of infection and mice were humanely sacrificed if ≥25% of initial weight was lost. Data shown is from one replicate. Significance was tested via log-rank sum test.

Wild type RML LVS uniformly protects C57Bl/6 mice against challenge with virulent *Francisella* whereas ATCC LVS does not (Griffin et al., 2015). We have established that an important difference between RML LVS and ATCC LVS is their acquisition of iron and subsequent sensitivity to ROS that is inversely correlated with protective efficacy. Since mutation of *bfr* in RML LVS led to a decrease in P_*fslA*_*-lacZ* reporter activity, increased sensitivity to H_2_O_2_, and decreased virulence we hypothesized that vaccination with RML LVS Δ*bfr* would provide poor protection against challenge with virulent *F. tularensis* SchuS4. Accordingly, 6 weeks after infection with the RML LVS Δ*bfr* mutant, we challenged all animals that survived with *F. tularensis* SchuS4 (Figure 7). As a control, mice that had been intranasally infected with 121 CFU of RML LVS (*n* = 3) were also challenged with 25 CFU of *F. tularensis* SchuS4. As expected all mice that had previously been infected with RML LVS survived SchuS4 challenge. In contrast, none of the mice previously infected with RML LVS Δ*bfr* strain were protected from the SchuS4 infection and all mice died within 8 days of challenge. Thus, while prior infection with RML LVS Δ*bfr* did extend the mean time to death, it provoked incomplete protection against subsequent infection with fully virulent SchuS4. Together this suggests that *Francisella* iron homeostasis and its interplay with host elements that control bacterial replication are important factors that contribute to both survival of primary infection and induction of adaptive immune responses *in vivo*.

## Discussion

*Francisella tularensis* is a highly pathogenic bacterium for which no effective licensed vaccine exists. Due to concerns that the extreme pathogenicity of the organism might lead to its intentional release there is increased interest in developing an effective vaccine for *F. tularensis* infections. A complication with the LVS is that the genetic mutations that attenuated LVS are undefined, leaving open the possibility that the strain could revert to full virulence. While LVS is unlikely to be reapproved for human vaccination, it is used extensively as a standard by which other candidate *Francisella* vaccines can be measured in animal models, as well as a tool to discover correlates of immunity to *F. tularensis*. Indeed, in a previous report it was found that the RML LVS biovar stimulated protective immunity in a mouse model of vaccine against virulent *F. tularensis* Schu S4 by inducing higher numbers of effector T cells (Griffin et al., 2015). Building on this observation, we have sought to identify the genetic differences between biovars of LVS and explore how they may affect the outcomes of virulence and immunity in mouse models of infection. In this report, we have demonstrated that there is a significant difference between the RML LVS and the Iowa LVS or ATCC LVS biovars in ferrous iron homeostasis.

Iron is one of the most abundant metals on the planet but is usually found in the environment in an insoluble oxidized state, which has required bacteria to develop mechanisms to scavenge iron from their local environment (Crichton and Pierre, 2001). The need for bacterial pathogens to acquire iron within a host environment presents additional challenges to the microorganism as they must also contend with host responses, which include mounting an immune response and sequestering iron, both of which can severely limit the ability of the pathogen to grow within the host (Cassat and Skaar, 2013). As expected, *F. tularensis* requires iron for productive infection of host cells, as mutants in ferrous and ferric iron uptake systems in *F. tularensis* LVS and *F. tularensis* Schu S4 were incapable of intracellular growth *in vitro* and were attenuated for virulence in mice (Perez and Ramakrishnan, 2014; Perez et al., 2016). Other work has shown that *F. tularensis* significantly increases expression of host iron-related genes, including transferrin receptor, presumably to increase the labile iron pools in the cytosol of infected host cells (Pan et al., 2010). Although *F. tularensis* requires iron for growth in a host, it appears to be unusual among bacterial pathogens in that its virulence may depend, in part, on maintaining optimally low levels of iron within its cytoplasm, as the highly virulent subsp. *tularensis* strains had approximately 4- to 5-fold less intracellular iron than the moderately virulent subsp. *holarctica* strains (Lindgren et al., 2011).

Consistent with this model of *Francisella* virulence, we have shown that the more virulent RML LVS biovar encodes a non-synonymous mutation in the *feoB* gene that confers phenotypes consistent with lower ferrous iron levels. The strain grows similarly to the Iowa and ATCC LVS biovars in standard propagation media, yet is restricted for growth when the iron concentrations are significantly reduced. This indicates that when extracellular iron levels become limiting, the RML LVS may not be as proficient at scavenging iron (via the *feoB* system) as Iowa LVS and ATCC LVS. Numerous labs have reported that expression of the *fsl* iron uptake operon genes are tightly regulated (repressed in high iron, induced in low iron) by iron concentrations in the media, so we used a P_*fslA*_-*lacZ* reporter as an indirect measure of relative ferrous iron levels between the various LVS biovars (Deng et al., 2006; Ramakrishnan et al., 2008; Lindgren et al., 2009). As with growth phenotypes, there were no detectable differences in P_*fslA*_*-lacZ* expression in growth media with normal iron concentrations; however, the RML LVS had increased reporter activity (consistent with lower intracellular iron levels) relative to the Iowa LVS and ATCC LVS after growth in iron limiting media. We probed *feoB* function of the various LVS strains further by performing functional complementation experiments. Using a *E. coli* Δ *feoB* mutant with a Fur-repressed *fhuF*-*lacZ* reporter as the complementation strain, we observed that the RML LVS *feoB* allele did not mediate repression of the *fhuF*-*lacZ* reporter (low intracellular iron), while both the Iowa LVS and ATCC LVS alleles did (high intracellular iron concentrations). Collectively, these experimental results provide compelling genetic evidence that the RML LVS likely has lower basal levels of intracellular ferrous iron than the Iowa LVS and ATCC LVS biovars. To our knowledge this is the first direct genetic evidence that explains differences in iron content between *F. tularensis* strains, though it is likely unique to LVS biovars. A significant contribution of this work is that it helps to provide a rationale for why virulent *F. tularensis* strains may carefully control their levels of intracellular iron as it appears that they balance the requirement of iron for growth against the effects of the Fenton reaction, which may induce oxidant damage to the bacteria that could expose the organisms to the host immune response (Binesse et al., 2015).

The low ferrous iron phenotypes exhibited by subsp. *tularensis* have been reported to correlate with resistance to the lethal effects of H_2_O_2_, so we sought to characterize the relative sensitivity of each LVS biovar *in vitro*. Indeed, the RML LVS exhibited nearly ten-fold more resistance to hydrogen peroxide than the Iowa LVS, which encodes a functional FeoB, and 100-fold or more resistance than the ATCC LVS. The latter strain is significantly more sensitive to H_2_O_2_ as it is competent for Fe^2+^ uptake through its functional FeoB, and because it encodes a Dyp peroxidase lacking 31 amino acids near the C-terminal portion of the protein. Mutation of the *dyp* gene has been shown previously to increase H_2_O_2_ sensitivity (Binesse et al., 2015), and a clean deletion of *dyp* in the Iowa LVS recapitulates this phenotype (unpublished data). We found that the RML LVS became significantly more sensitive to H_2_O_2_ by introducing a functional *feoB* allele under constitutive expression on a plasmid; the H_2_O_2_ sensitivity was significantly lower when the nonfunctional RML allele was introduced into the strain. We further demonstrated that the increased H_2_O_2_ lethality associated with overexpression of a functional *feoB* was not unique to the RML LVS genetic background, as an Iowa LVS Δ*feoB* mutant complemented with the mutant allele was more resistant to H_2_O_2_ than that complemented with the functional allele. These experiments are significant, as the strains only differed by a single nucleotide in *feoB*, yet exhibited nearly ten-fold differences in their sensitivities to H_2_O_2_, irrespective of genetic background. In sum, these experiments strongly suggest that RML LVS is better at avoiding Fe^2+^-associated toxicity in the context of the host environment virtue due to its lower functioning FeoB system.

In addition to characterizing the *feoB* D471Y mutation in RML LVS, we also characterized a mutant lacking the putative iron storage protein bacterioferritin. Bacterioferritin is known to oxidize ferrous iron and store the ferric oxide mineral in the hollow cavity formed by a 24-mer sphere in other organisms (Honarmand Ebrahimi et al., 2015). We reasoned that a *F. tularensis* RML LVS mutant lacking bacterioferritin may be unable to sequester free Fe^2+^ as effectively as the wild type strain, and that this would be reflected in iron-related gene expression. The P_*fslA*_*-lacZ* reporter indeed showed a modest decrease in expression in the Δ*bfr* mutant, indicating that the strain may have more Fe^2+^ available to interact with Fur and repress expression of the reporter. This result is consistent with the hypothesis that *F. tularensis* LVS uses bacterioferritin to alter the pools of intracellular Fe^2+^ levels by sequestration. The P_*fslA*_*-lacZ* reporter expression decreased by only ~20% suggesting that the bacteria may utilize other iron storage mechanisms as well. As the iron-regulated reporter indicated that the Δ*bfr* mutant may have increased levels of intracellular ferrous iron, we hypothesized that the strain would be more sensitive to H_2_O_2_. We found that the Δ*bfr* mutant had approximately ~90% reduction in viability relative to the parental strain, indicating that bacterioferritin has a role in resistance to oxidative stress in *F. tularensis* LVS.

As the plasmid-based complementation strategy for the Iowa LVS Δ*feoB* was unsuitable for *in vivo* studies, we utilized the RML LVS Δ*bfr* mutant as an alternative for proof of principle, given the lower P_*fslA*_*-lacZ* reporter activity and the increased sensitivity to H_2_O_2_ of this mutant. Intranasal infections of mice confirmed that bacterioferritin was required for full virulence of the strain, with the LD_50_ of the mutant estimated to be ~1,500 CFU, nearly an eight-fold increase relative to the parental strain (<200 CFU). Additionally, the mice exhibited differences in the severity of disease, as mice infected with 133 CFU of RML LVS lost considerable weight during the infection, approximately twenty-three percent on average. In contrast, the mice infected with 215 CFU of the Δ*bfr* mutant lost significantly less body weight during infection (~12%), even though the dose was almost two times higher. Only at a ten-fold higher dose of the Δ*bfr* mutant did the mice lose similar amounts of weight as those infected with the lowest dose of the parent strain.

As the RML LVS has been shown to be more effective at stimulating a protective response to challenge with *F. tularensis* Schu S4 than the more H_2_O_2_ sensitive biovar of LVS (ATCC), we hypothesized that loss of H_2_O_2_ resistance would render it a poor vaccine strain. Consistent with our hypothesis, the RML LVS Δ*bfr* did not stimulate robust protection in mice infected with this strain, as all succumbed from Schu S4 infection by day eight. These data suggest that H_2_O_2_ resistance may contribute to the efficacy of the RML LVS as a vaccine against tularemia in mice; however, it is possible that other interpretations of the data are valid. For example, the bacterioferritin protein is known to stimulate antibody production and T cell proliferation in LVS immunized mice, however it isn't clear that antibody responses are effective at controlling infection with virulent *F. tularensis* Schu S4 (Bakshi et al., 2008). In *in vitro* studies, *F. tularensis* Schu S4 is able to bind the active form of the host protease plasmin, which can cleave *Francisella* specific antibodies to avoid the ensuing bactericidal and inflammatory consequences of phagocytosis via antibody mediated opsonization (Crane et al., 2009). Furthermore, introduction of monoclonal antibodies against *Francisella* bacterioferritin into mice did not provide protection against lethal LVS challenge, even though other antibodies can be highly effective at protecting against this strain (Cole et al., 2009; Savitt et al., 2009). Taken together, these observations suggest that the lack of a bacterioferritin-specific antibody response is not likely to explain the poor efficacy of the RML LVS Δ*bfr* at protecting against Schu S4.

This work is significant for several reasons. The observation that the RML LVS is better able to induce host immunity than other LVS strains and has phenotypes consistent with relatively lower levels of free ferrous iron indicates that there is an important inverse correlation between the two. This finding is of particular importance for research groups using *F. tularensis* LVS as a model organism for studying tularemia. Care should be taken as lab workers plate LVS cultures during routine work to not over-passage LVS and/or inadvertently select for isolates that carry mutations that lead to higher acquisition of iron. This may have the unintended consequence of working with LVS isolates that have reduced immunogenicity and/or virulence. In addition, our findings highlight further the virulence strategy of *F. tularensis* which appears to be optimizing its ability to grow while avoiding, as much as possible, its visibility to the host's immune surveillance systems. Presumably, this contributes significantly to the extreme virulence of this bacterial pathogen. Finally, we believe that this work provides new information that should help to guide efforts to design a rational vaccine for tularemia.

## Ethics statement

All experiments using recombinant DNA techniques or reagents with *F. tularensis* LVS were approved by the Institutional Biosafety Committee. All animals were handled in strict accordance with good animal practice as defined by the relevant national and/or local animal welfare bodies, and all animal work was approved by the University of Iowa Animal Care and Use committee (ACURF #1305086). Guidelines provided by the NIH were followed in all experimentation. The University of Iowa is PHS assured.

## Author contributions

JF designed and performed a majority of experiments and was responsible for writing the manuscript. DC and TW designed and performed experiments in which LVS strains were used to infect macrophages and stimulate cytokine production. They also participated in editing the manuscript. CM performed bioinformatics analysis of the genomic sequences of strains and was responsible for editing the manuscript. CB oversaw experiments in her lab, she is responsible for the first observation of virulence differences in LVS strains and she edited and helped to write the manuscript. BJ oversaw research work performed by JF in his lab, is responsible for the final state of the manuscript and is the corresponding author.

### Conflict of interest statement

The authors declare that the research was conducted in the absence of any commercial or financial relationships that could be construed as a potential conflict of interest.

## References

[B1] Andrews S. C. Robinson A. K. Rodriguez-Quinones F. (2003). Bacterial iron homeostasis. FEMS Microbiol. Rev. 27, 215–237. 10.1016/S0168-6445(03)00055-X 12829269

[B2] Aranda J. Cortés M. P. Garrido M. E. Fittipaldi N. Llagostera M. Gottschalk M.. (2009). Contribution of the FeoB transporter to Streptococcus suis virulence. Int. Microbiol. 12, 137–143. 10.2436/20.1501.01.91 19784934

[B3] Ashkenazy H. Erez E. Martz E. Pupko T. Ben-Tal N. (2010). ConSurf 2010: calculating evolutionary conservation in sequence and structure of proteins and nucleic acids. Nucleic Acids Res. 38, W529–W533. 10.1093/nar/gkq399 20478830 PMC2896094

[B4] Bakshi C. S. Malik M. Mahawar M. Kirimanjeswara G. S. Hazlett K. R. Palmer L. E.. (2008). An improved vaccine for prevention of respiratory tularemia caused by *Francisella tularensis* SchuS4 strain. Vaccine 26, 5276–5288. 10.1016/j.vaccine.2008.07.051 18692537 PMC2652725

[B5] Bakshi C. S. Malik M. Regan K. Melendez J. A. Metzger D. W. Pavlov V. M.. (2006). Superoxide dismutase B gene (sodB)-deficient mutants of *Francisella tularensis* demonstrate hypersensitivity to oxidative stress and attenuated virulence. J. Bacteriol. 188, 6443–6448. 10.1128/JB.00266-06 16923916 PMC1595384

[B6] Binesse J. Lindgren H. Lindgren L. Conlan W. Sjostedt A. (2015). Roles of reactive oxygen species-degrading enzymes of *Francisella tularensis* SCHU S4. Infect. Immun. 83, 2255–2263. 10.1128/IAI.02488-14 25802058 PMC4432764

[B7] Bou-Abdallah F. Lewin A. C. Le Brun N. E. Moore G. R. Chasteen N. D. (2002). Iron detoxification properties of *Escherichia coli* bacterioferritin. Attenuation of oxyradical chemistry. J. Biol. Chem. 277, 37064–37069. 10.1074/jbc.M205712200 12124394

[B8] Buchan B. W. McCaffrey R. L. Lindemann S. R. Allen L. A. Jones B. D. (2009). Identification of migR, a regulatory element of the *Francisella tularensis* live vaccine strain iglABCD virulence operon required for normal replication and trafficking in macrophages. Infect. Immun. 77, 2517–2529. 10.1128/IAI.00229-09 19349423 PMC2687360

[B9] Buchan B. W. McLendon M. K. Jones B. D. (2008). Identification of differentially regulated *francisella tularensis* genes by use of a newly developed Tn5-based transposon delivery system. Appl. Environ. Microbiol. 74, 2637–2645. 10.1128/AEM.02882-07 18344342 PMC2394869

[B10] Cartron M. L. Maddocks S. Gillingham P. Craven C. J. Andrews S. C. (2006). Feo–transport of ferrous iron into bacteria. Biometals 19, 143–157. 10.1007/s10534-006-0003-2 16718600

[B11] Cassat J. E. Skaar E. P. (2013). Iron in infection and immunity. Cell Host Microbe 13, 509–519. 10.1016/j.chom.2013.04.010 23684303 PMC3676888

[B12] Cole L. E. Yang Y. Elkins K. L. Fernandez E. T. Qureshi N. Shlomchik M. J.. (2009). Antigen-specific B-1a antibodies induced by *Francisella tularensis* LPS provide long-term protection against *F. tularensis* LVS challenge. Proc. Natl. Acad. Sci. U.S.A. 106, 4343–4348. 10.1073/pnas.0813411106 19251656 PMC2657382

[B13] Crane D. D. Bauler T. J. Wehrly T. D. Bosio C. M. (2014). Mitochondrial ROS potentiates indirect activation of the AIM2 inflammasome. Front. Microbiol. 5:438. 10.3389/fmicb.2014.00438 25191316 PMC4138581

[B14] Crane D. D. Warner S. L. Bosio C. M. (2009). A novel role for plasmin-mediated degradation of opsonizing antibody in the evasion of host immunity by virulent, but not attenuated, *Francisella tularensis*. J. Immunol. 183, 4593–4600. 10.4049/jimmunol.0901655 19752236 PMC2748154

[B15] Crichton R. R. Pierre J. L. (2001). Old iron, young copper: from Mars to Venus. Biometals 14, 99–112. 10.1023/A:1016710810701 11508852

[B16] Deng K. Blick R. J. Liu W. Hansen E. J. (2006). Identification of *Francisella tularensis* genes affected by iron limitation. Infect. Immun. 74, 4224–4236. 10.1128/IAI.01975-05 16790797 PMC1489736

[B17] Edwards J. A. Rockx-Brouwer D. Nair V. Celli J. (2010). Restricted cytosolic growth of *Francisella tularensis* subsp. tularensis by IFN-gamma activation of macrophages. Microbiology 156, 327–339. 10.1099/mic.0.031716-0 19926654 PMC2890092

[B18] Eigelsbach H. T. Downs C. M. (1961). Prophylactic effectiveness of live and killed tularemia vaccines. I. Production of vaccine and evaluation in the white mouse and guinea pig. J. Immunol. 87, 415–425. 13889609

[B19] Griffin A. J. Crane D. D. Wehrly T. D. Bosio C. M. (2015). Successful protection against tularemia in C57BL/6 mice is correlated with expansion of *Francisella tularensis*-specific effector T cells. Clin. Vaccine Immunol. 22, 119–128. 10.1128/CVI.00648-14 25410207 PMC4278928

[B20] Hantke K. (2003). Is the bacterial ferrous iron transporter FeoB a living fossil? Trends Microbiol. 11, 192–195. 10.1016/S0966-842X(03)00100-8 12781516

[B21] Honarmand Ebrahimi K. Hagedoorn P. L. Hagen W. R. (2015). Unity in the biochemistry of the iron-storage proteins ferritin and bacterioferritin. Chem. Rev. 115, 295–326. 10.1021/cr5004908 25418839

[B22] Imlay J. A. (2013). The molecular mechanisms and physiological consequences of oxidative stress: lessons from a model bacterium. Nat. Rev. Microbiol 11, 443–454. 10.1038/nrmicro3032 23712352 PMC4018742

[B23] Kammler M. Schon C. Hantke K. (1993). Characterization of the ferrous iron uptake system of *Escherichia coli*. J. Bacteriol. 175, 6212–6219. 10.1128/jb.175.19.6212-6219.1993 8407793 PMC206716

[B24] Kim H. Lee H. Shin D. (2012). The FeoA protein is necessary for the FeoB transporter to import ferrous iron. Biochem. Biophys. Res. Commun. 423, 733–738. 10.1016/j.bbrc.2012.06.027 22705302

[B25] Landau M. Mayrose I. Rosenberg Y. Glaser F. Martz E. Pupko T.. (2005). ConSurf 2005: the projection of evolutionary conservation scores of residues on protein structures. Nucleic Acids Res. 33, W299–W302. 10.1093/nar/gki370 15980475 PMC1160131

[B26] Langmead B. Salzberg S. L. (2012). Fast gapped-read alignment with Bowtie 2. Nat. Methods 9, 357–359. 10.1038/nmeth.1923 22388286 PMC3322381

[B27] Lau C. K. Ishida H. Liu Z. Vogel H. J. (2013). Solution structure of Escherichia coli FeoA and its potential role in bacterial ferrous iron transport. J. Bacteriol. 195, 46–55. 10.1128/JB.01121-12 23104801 PMC3536175

[B28] Lindemann S. R. Peng K. Long M. E. Hunt J. R. Apicella M. A. Monack D. M.. (2011). *Francisella tularensis* Schu S4 O-antigen and capsule biosynthesis gene mutants induce early cell death in human macrophages. Infect. Immun. 79, 581–594. 10.1128/IAI.00863-10 21078861 PMC3028865

[B29] Lindgren H. Honn M. Golovlev I. Kadzhaev K. Conlan W. Sjöstedt A. (2009). The 58-kilodalton major virulence factor of *Francisella tularensis* is required for efficient utilization of iron. Infect. Immun. 77, 4429–4436. 10.1128/IAI.00702-09 19651867 PMC2747937

[B30] Lindgren H. Honn M. Salomonsson E. Kuoppa K. Forsberg Å. Sjöstedt A. (2011). Iron content differs between *Francisella tularensis* subspecies *tularensis* and subspecies holarctica strains and correlates to their susceptibility to H_2_O_2_-induced killing. Infect. Immun. 79, 1218–1224. 10.1128/IAI.01116-10 21189323 PMC3067496

[B31] Lindgren H. Lindgren L. Golovliov I. Sjostedt A. (2015). Mechanisms of heme utilization by *Francisella tularensis*. PLoS ONE 10:e0119143. 10.1371/journal.pone.0119143 25756756 PMC4355490

[B32] Lindgren H. Shen H. Zingmark C. Golovliov I. Conlan W. Sjöstedt A. (2007). Resistance of *Francisella tularensis* strains against reactive nitrogen and oxygen species with special reference to the role of KatG. Infect. Immun. 75, 1303–1309. 10.1128/IAI.01717-06 17210667 PMC1828546

[B33] Llewellyn A. C. Jones C. L. Napier B. A. Bina J. E. Weiss D. S. (2011). Macrophage replication screen identifies a novel Francisella hydroperoxide resistance protein involved in virulence. PLoS ONE 6:e24201. 10.1371/journal.pone.0024201 21915295 PMC3167825

[B34] Ma Z. Banik S. Rane H. Mora V. T. Rabadi S. M. Doyle C. R.. (2014). EmrA1 membrane fusion protein of *Francisella tularensis* LVS is required for resistance to oxidative stress, intramacrophage survival and virulence in mice. Mol. Microbiol. 91, 976–995. 10.1111/mmi.12509 24397487 PMC4097035

[B35] Marlovits T. C. Haase W. Herrmann C. Aller S. G. Unger V. M. (2002). The membrane protein FeoB contains an intramolecular G protein essential for Fe(II) uptake in bacteria. Proc. Natl. Acad. Sci. U.S.A. 99, 16243–16248. 10.1073/pnas.242338299 12446835 PMC138596

[B36] McCaffrey R. L. Schwartz J. T. Lindemann S. R. Moreland J. G. Buchan B. W. Jones B. D.. (2010). Multiple mechanisms of NADPH oxidase inhibition by type A and type B *Francisella tularensis*. J. Leukoc. Biol. 88, 791–805. 10.1189/jlb.1209811 20610796 PMC2974429

[B37] McKenna A. Hanna M. Banks E. Sivachenko A. Cibulskis K. Kernytsky A.. (2010). The genome analysis toolkit: a MapReduce framework for analyzing next-generation DNA sequencing data. Genome Res. 20, 1297–1303. 10.1101/gr.107524.110 20644199 PMC2928508

[B38] Melillo A. A. Bakshi C. S. Melendez J. A. (2010). *Francisella tularensis* antioxidants harness reactive oxygen species to restrict macrophage signaling and cytokine production. J. Biol. Chem. 285, 27553–27560. 10.1074/jbc.M110.144394 20558723 PMC2934622

[B39] Melillo A. A. Mahawar M. Sellati T. J. Malik M. Metzger D. W. Melendez J. A.. (2009). Identification of *Francisella tularensis* live vaccine strain CuZn superoxide dismutase as critical for resistance to extracellularly generated reactive oxygen species. J. Bacteriol. 191, 6447–6456. 10.1128/JB.00534-09 19684141 PMC2753026

[B40] Nagy T. A. Moreland S. M. Detweiler C. S. (2014). Salmonella acquires ferrous iron from haemophagocytic macrophages. Mol. Microbiol. 93, 1314–1326. 10.1111/mmi.12739 25081030 PMC4160465

[B41] Naikare H. Palyada K. Panciera R. Marlow D. Stintzi A. (2006). Major role for FeoB in *Campylobacter jejuni* ferrous iron acquisition, gut colonization, and intracellular survival. Infect. Immun. 74, 5433–5444. 10.1128/IAI.00052-06 16988218 PMC1594910

[B42] Pan X. Tamilselvam B. Hansen E. J. Daefler S. (2010). Modulation of iron homeostasis in macrophages by bacterial intracellular pathogens. BMC Microbiol. 10:64. 10.1186/1471-2180-10-64 20184753 PMC2838877

[B43] Parrow N. L. Fleming R. E. Minnick M. F. (2013). Sequestration and scavenging of iron in infection. Infect. Immun. 81, 3503–3514. 10.1128/IAI.00602-13 23836822 PMC3811770

[B44] Perez N. Johnson R. Sen B. Ramakrishnan G. (2016). Two parallel pathways for ferric and ferrous iron acquisition support growth and virulence of the intracellular pathogen Francisella tularensis Schu S4. Microbiologyopen 5, 453–468. 10.1002/mbo3.342 26918301 PMC4905997

[B45] Perez N. M. Ramakrishnan G. (2014). The reduced genome of the *Francisella tularensis* live vaccine strain (LVS) encodes two iron acquisition systems essential for optimal growth and virulence. PLoS ONE 9:e93558. 10.1371/journal.pone.0093558 24695402 PMC3973589

[B46] Rabadi S. M. Sanchez B. C. Varanat M. Ma Z. Catlett S. V. Melendez J. A.. (2015). Antioxidant defenses of Francisella tularensis modulate macrophage function and production of proinflammatory cytokines. J. Biol. Chem. 291, 5009–5021. 10.1074/jbc.M115.681478 26644475 PMC4777838

[B47] Ramakrishnan G. Meeker A. Dragulev B. (2008). fslE is necessary for siderophore-mediated iron acquisition in *Francisella tularensis* Schu S4. J. Bacteriol. 190, 5353–5361. 10.1128/JB.00181-08 18539739 PMC2493265

[B48] Ramakrishnan G. Sen B. (2014). The FupA/B protein uniquely facilitates transport of ferrous iron and siderophore-associated ferric iron across the outer membrane of *Francisella tularensis* live vaccine strain. Microbiology 160, 446–457. 10.1099/mic.0.072835-0 24307666 PMC3919536

[B49] Ramond E. Gesbert G. Rigard M. Dairou J. Dupuis M. Dubail I.. (2014). Glutamate utilization couples oxidative stress defense and the tricarboxylic acid cycle in Francisella phagosomal escape. PLoS Pathog. 10:e1003893. 10.1371/journal.ppat.1003893 24453979 PMC3894225

[B50] Reed L. J. Muench H. (1938). A simple method of estimating fifty percent endpoints. Am. J. Hygiene 27, 493–497.

[B51] Robey M. Cianciotto N. P. (2002). *Legionella pneumophila* feoAB promotes ferrous iron uptake and intracellular infection. Infect. Immun. 70, 5659–5669. 10.1128/IAI.70.10.5659-5669.2002 12228295 PMC128349

[B52] Rohmer L. Fong C. Abmayr S. Wasnick M. Larson Freeman T. J. Radey M.. (2007). Comparison of *Francisella tularensis* genomes reveals evolutionary events associated with the emergence of human pathogenic strains. Genome Biol. 8:R102. 10.1186/gb-2007-8-6-r102 17550600 PMC2394750

[B53] Rohmer L. Brittnacher M. Svensson K. Buckley D. Haugen E. Zhou Y.. (2006). Potential source of *Francisella tularensis* live vaccine strain attenuation determined by genome comparison. Infect. Immun. 74, 6895–6906. 10.1128/IAI.01006-06 17000723 PMC1698093

[B54] Salomonsson E. Kuoppa K. Forslund A. L. Zingmark C. Golovliov I. Sjöstedt A.. (2009). Reintroduction of two deleted virulence loci restores full virulence to the live vaccine strain of *Francisella tularensis*. Infect. Immun. 77, 3424–3431. 10.1128/IAI.00196-09 19506014 PMC2715654

[B55] Savitt A. G. Mena-Taboada P. Monsalve G. Benach J. L. (2009). *Francisella tularensis* infection-derived monoclonal antibodies provide detection, protection, and therapy. Clin. Vaccine Immunol. 16, 414–422. 10.1128/CVI.00362-08 19176692 PMC2650858

[B56] Schulert G. S. McCaffrey R. L. Buchan B. W. Lindemann S. R. Hollenback C. Jones B. D.. (2009). *Francisella tularensis* genes required for inhibition of the neutrophil respiratory burst and intramacrophage growth identified by random transposon mutagenesis of strain LVS. Infect. Immun. 77, 1324–1336. 10.1128/IAI.01318-08 19204089 PMC2663180

[B57] Shakerley N. L. Chandrasekaran A. Trebak M. Miller B. A. Melendez J. A. (2015). Francisella tularensis catalase restricts immune function by impairing TRPM2 channel activity. J Biol. Chem. 291, 3871–3881. 10.1074/jbc.M115.706879 26679996 PMC4759167

[B58] Su J. Yang J. Zhao D. Kawula T. H. Banas J. A. Zhang J. R. (2007). Genome-wide identification of *Francisella tularensis* virulence determinants. Infect. Immun. 75, 3089–3101. 10.1128/IAI.01865-06 17420240 PMC1932872

[B59] Su Y. C. Chin K. H. Hung H. C. Shen G. H. Wang A. H. Chou S. H. (2010). Structure of *Stenotrophomonas maltophilia* FeoA complexed with zinc: a unique prokaryotic SH3-domain protein that possibly acts as a bacterial ferrous iron-transport activating factor. Acta Crystallogr. F Struct. Biol. Cryst. Commun. 66, 636–642. 10.1107/S1744309110013941 20516589 PMC2882759

[B60] Sullivan J. T. Jeffery E. F. Shannon J. D. Ramakrishnan G. (2006). Characterization of the siderophore of *Francisella tularensis* and role of fslA in siderophore production. J. Bacteriol. 188, 3785–3795. 10.1128/JB.00027-06 16707671 PMC1482922

[B61] Thomas-Charles C. A. Zheng H. Palmer L. E. Mena P. Thanassi D. G. Furie M. B.. (2013). FeoB-mediated uptake of iron by *Francisella tularensis*. Infect. Immun. 81, 2828–2837. 10.1128/IAI.00170-13 23716605 PMC3719576

[B62] Troxell B. Hassan H. M. (2013). Transcriptional regulation by Ferric Uptake Regulator (Fur) in pathogenic bacteria. Front. Cell. Infect. Microbiol. 3:59. 10.3389/fcimb.2013.00059 24106689 PMC3788343

[B63] Velayudhan J. Hughes N. J. McColm A. A. Bagshaw J. Clayton C. L. Andrews S. C.. (2000). Iron acquisition and virulence in *Helicobacter pylori*: a major role for FeoB, a high-affinity ferrous iron transporter. Mol. Microbiol. 37, 274–286. 10.1046/j.1365-2958.2000.01987.x 10931324

[B64] Weaver E. A. Wyckoff E. E. Mey A. R. Morrison R. Payne S. M. (2013). FeoA and FeoC are essential components of the Vibrio cholerae ferrous iron uptake system, and FeoC interacts with FeoB. J. Bacteriol. 195, 4826–4835. 10.1128/JB.00738-13 23955009 PMC3807486

[B65] Wehrly T. D. Chong A. Virtaneva K. Sturdevant D. E. Child R. Edwards J. A.. (2009). Intracellular biology and virulence determinants of Francisella tularensis revealed by transcriptional profiling inside macrophages. Cell. Microbiol. 11, 1128–1150. 10.1111/j.1462-5822.2009.01316.x 19388904 PMC2746821

[B66] Zubay G. Morse D. E. Schrenk W. J. Miller J. H. (1972). Detection and isolation of the repressor protein for the tryptophan operon of *Escherichia coli*. Proc. Natl. Acad. Sci. U.S.A. 69, 1100–1103. 10.1073/pnas.69.5.1100 4338582 PMC426639

